# Assessment of safety profile and anti-obesity effects of innovative plant-based food formulations in a zebrafish diet-induced obesity model

**DOI:** 10.3389/fnut.2026.1867646

**Published:** 2026-06-12

**Authors:** Justyna Magdalena Hermanowicz, Sandra Budziak, Renata Markiewicz-Żukowska, Anna Puścion-Jakubik, Monika Grabia-Lis, Justyna Moskwa, Sylwia Katarzyna Naliwajo, Michał Tomczyk, Arkadiusz Surażyński, Anna Pryczynicz, Dariusz Pawlak, Katarzyna Socha

**Affiliations:** 1 Department of Clinical Pharmacy, Faculty of Pharmacy with the Division of Laboratory Medicine, Medical University of Bialystok, Białystok, Poland; 2 Department of Bromatology, Faculty of Pharmacy with the Division of Laboratory Medicine, Medical University of Bialystok, Białystok, Poland; 3 Department of Biology and Pharmacognosy, Faculty of Pharmacy with the Division of Laboratory Medicine, Medical University of Bialystok, Białystok, Poland; 4 Department of Medicinal Chemistry, Faculty of Pharmacy with the Division of Laboratory Medicine, Medical University of Bialystok, Białystok, Poland; 5 Department of General Pathomorphology, Faculty of Pharmacy of Health Science, Medical University of Bialystok, Białystok, Poland; 6 Department of Pharmacodynamics, Faculty of Pharmacy with the Division of Laboratory Medicine, Medical University of Bialystok, Białystok, Poland

**Keywords:** functional food, glucose, metabolic disorders, obesity, plant-based products, zebrafish

## Abstract

**Introduction:**

Obesity is a complex metabolic disorder associated with impaired glucose, lipid metabolism and excessive visceral fat accumulation. Therefore, the development of effective dietary strategies, including functional foods, has attracted increasing interest. This study aimed to evaluate the safety and the metabolic effects of three food formulations (F1, F2, and F3) using a zebrafish model.

**Methods:**

The safety profile of the complex of active substances was assessed in zebrafish embryos and larvae by evaluating survival, hatching rate, spontaneous movement, morphological abnormalities, and cardiac function. To investigate metabolic effects, diet-induced obesity was established in adult zebrafish.

**Results:**

Supplementation with F1, F2, or F3 did not toxicity or developmental defects at early life stages. It resulted in significant reductions in body weight, glucose, triglycerides, and total cholesterol levels compared to obese controls. Additionally, formulations reduced visceral fat content, while F1 and F2 also significantly decreased subcutaneous fat.

**Discussion:**

These findings demonstrate that the tested food formulations are safe and exert beneficial effects on metabolic parameters and fat accumulation in zebrafish. The results suggest their potential as functional dietary interventions contributing to the amelioration of diet-induced obesity-related metabolic dysfunction.

## 1 Introduction

Obesity is a chronic, complex disease that affects an increasing number of people, reaching epidemic proportions worldwide. It increases the risk of developing metabolic disorders such as type 2 diabetes and dyslipidemia; however, metabolic dysregulation may also be a crucial factor contributing to the development of obesity (1, 2). Visceral fat enlargement is strongly associated with the development and progression of metabolic and cardiovascular diseases in obese individuals, and correlates with insulin resistance, which is commonly observed in these patients (3). Furthermore, obesity is associated with alterations in glucose regulation and contributes to glucose intolerance (4). Dyslipidemia is another metabolic disorder highly prevalent in obese individuals and is linked to abnormal levels of cholesterol and triglycerides (5, 6). These metabolic disturbances underline the importance of dietary interventions, including functional foods, as supportive approaches for the prevention and treatment of obesity and its metabolic complications (2). Functional foods are regarded as products that provide health benefits beyond their basic nutritional value due to the presence of bioactive compounds (7). In the context of obesity, functional foods may help prevent excessive weight gain and metabolic disturbances by supporting energy balance, glucose regulation, and lipid metabolism. These products often include low-fat or low-sugar formulations as well as ingredients of plant or animal origin with potential beneficial metabolic effects (8).

Due to their genetic, anatomical, and physiological similarities to humans, zebrafish have become an important vertebrate model organism used in numerous areas of biomedical research (9–11). They are widely applied in developmental biology, toxicology, drug discovery, human genetics, and studies investigating the mechanisms of various human diseases. In recent years, this model has also attracted growing interest in research on metabolic disorders, including obesity (12). One of the major advantages of zebrafish is the optical transparency of their larval stage, which allows direct visualization of lipid accumulation throughout the whole organism using staining techniques such as Nile red (13). Additionally, zebrafish exhibit lipid metabolism and adipogenesis processes that are highly comparable to those observed in mammals. Importantly, obesity can be effectively modelled in adult zebrafish (14). Overfeeding-induced obesity in this species results in metabolic alterations resembling those seen in humans, including hypertriglyceridemia and hepatic steatosis, and involves similar underlying pathophysiological mechanisms. These characteristics make zebrafish a valuable experimental system for studying the development of obesity as well as for assessing the potential effects of functional foods and bioactive compounds in its prevention and treatment (15–17).

A multi-component complex of active substances (AS) was developed, comprising quercetin, rutin, betaine, zinc, and mulberry extract and then incorporated into three nutritional matrices (formulation F1, F2, F3). The selection of these ingredients was based on a literature review, indicating their documented metabolic, antioxidant, and anti-inflammatory potential, as well as the potential for complementary and synergistic effects. Quercetin and rutin were selected for their potent antioxidant properties and potential modulation of oxidative stress and inflammatory processes associated with metabolic disorders. Betaine was included as a compound supporting lipid metabolism and liver function, while zinc was included as an important enzymatic cofactor involved in the regulation of glucose metabolism and antioxidant processes. Mulberry extract was included for its potential to support glycemic control through its influence on carbohydrate metabolism. The novelty of these formulations lies not only in the use of these bioactive substances, but above all in their targeted combination within three distinct nutritional matrices, designed to provide comprehensive support for patients with metabolic disorders. The developed recipes represent an approach that integrates ingredients with different mechanisms of action, aimed at reducing oxidative stress, supporting glucose and lipid metabolism, and improving the functional value of the product.

The aim of our study was to evaluate the safety and efficacy of the developed prototypes using a zebrafish model. The safety profile was assessed in embryos and larvae. In the diet-induced obesity (DIO) model, glycemia and lipid profile parameters were analyzed. Adipose tissue content was evaluated using a fluorescence-based technique, while histopathological assessment was performed to measure the surface area of the largest adipocytes in both subcutaneous and visceral adipose tissue.

## 2 Materials and methods

### 2.1 Materials

Preparation of the three novel experimental formulations (F1–F3) followed a standardized protocol to ensure dietary homogeneity. Initially, all raw materials were micronized to achieve a uniform particle size of 700 μm. The components were then precisely weighed to constitute the final experimental additive. A complex of active substances (AS) was initially formulated, comprising white mulberry extract (47.06%; standardized to ≥1.4% 1-deoxynojirimycin), betaine anhydrous (27.43%; 99.7% betaine), quercetin extract (19.59%; standardized to 98% quercetin), rutin extract (5.88%; standardized to 95% rutin), and zinc citrate (0.05%; corresponding to 1.5672 mg of elemental zinc). This formulation was integrated into three distinct dietary matrices. Formulation no. 1 (F1) consisted of freeze-dried fruits (bilberry and lemon) and fruit juices (bilberry, apple, and red beet), a composition of dietary fibers (psyllium husk, inulin, glucomannan), and extracts (elderberry fruit extract containing at least 5% anthocyanins and 7.5% polyphenolic compounds; rosehip fruit extract standardized to at least 70% natural vitamin C, as well as stevia extract containing 98% rebaudioside A). Formulation no. 2 (F2) contained freeze-dried vegetables and fruits (tomato, zucchini, onion), flaxseed, buckwheat flakes, chickpea protein, tomato concentrate, Brazil nuts, and spices (salt, pepper, turmeric, onion, paprika, garlic, tomato, nutritional yeast flakes, parsley leaf), as well as goat’s rue extract at an E/D ratio of 4/1. Formulation no. 3 (F3) included such ingredients as freeze-dried and dried fruits (dates, white mulberry, strawberries, plum, orange), oat flakes, plant proteins (chickpea and sunflower seed), nuts (Brazil nuts and almonds), carob, inulin, rapeseed oil, and acerola extract standardized to at least 25% vitamin C. Each mixture was incorporated into the basal commercial fish feed at a 10% (w/w) inclusion rate (90% basal diet to 10% experimental product). To ensure uniform distribution of the active compounds, the final diet underwent rigorous mechanical homogenization before administration.

### 2.2 Methods

#### 2.2.1 Zebrafish husbandry

Embryos were obtained through natural mating from multiple breeding pairs. From 5-day post-fertilization (dpf), larvae were maintained in a recirculating water system at a temperature of 28 °C ± 1 °C and pH 6.5–8.5, under a 14/10-h light/dark cycle, in 5 L tanks at a density of approximately 30 larvae per litre. It should be noted that zebrafish embryos up to 5 dpf feed on substances derived from the yolk, therefore they do not require feeding, and studies using them are classified as *in vitro* studies. Zebrafish larvae older than 5 dpf were fed a standard diet depending on their developmental age until the beginning of the experiments. Animals were randomly assigned to the experimental groups prior to the initiation of the experimental protocol. In addition, investigators performing analyses were blinded to the group allocation during data collection and analysis in order to minimize potential bias. All animals were euthanized after completion of the experimental procedures by overdose of tricaine methanesulfonate (300 mg/L), according to Directive 2010/63/EU.

#### 2.2.2 Zebrafish toxicity evaluation

The safety assessment of the AS was performed based on the OECD 236 guideline (FET - Fish Embryo Acute Toxicity). In accordance with Gutierrez-Lover’s observations, to properly assess the safety profile, toxicity studies in the Zebrafish model should be conducted at two development stages (18). Freshly fertilized AB strain zebrafish embryos (0–2 h post-fertilization, hpf) showing normal development, as well as 72 hpf larvae, were placed in 24-well plates containing standard E3 medium along with 100 μg/mL complex of active substances (AS). Embryos were monitored at 24, 48, 72, and 96 h of exposure using a stereomicroscope equipped with a camera. All experiments were conducted in triplicate, with 20 embryos assigned to each group. Every 24 h, up to four key endpoints indicating mortality were assessed: coagulation of fertilized eggs, absence of somite formation, failure of the tail bud to detach from the yolk sac, and absence of a heartbeat. Additionally, spontaneous movement at 24 h and hatching rates at 48, 72, and 96 h were evaluated. At 96 h, further developmental parameters—including heart rate and total body length—as well as morphological abnormalities such as pericardial edema, yolk sac edema, tail curvature, impaired somite formation, and scoliosis, were recorded. Experiments were performed in 3 independent replicates.

#### 2.2.3 Zebrafish microinjection

Additionally, to assess the safety of the test products, 48 hpf embryos were divided into 2 groups (*n* = 60/group), anesthetized by placement in 40 mg/L ethyl 3-aminobenzoate methanesulfonate tricaine (until loss of equilibrium and absence of response to gentle tactile stimulation typically within 2–5 min), and injected with 8 nL of E3 (fish water) – control group and 100 μg/mL complex of active substances (AS) (19). Microinjection into a specific location in the yolk was performed under a microscope using a microinjector. Injected animals were monitored after 5 h and subsequently every 24 h to assess mortality and developmental defects. All animals were euthanized by overdose of tricaine methanesulfonate (300 mg/L), according to directive 2010/63/EU. Death was confirmed by cessation of opercular movements, absence of cardiac activity, and lack of reflex responses.

#### 2.2.4 Zebrafish obesogenic test (ZOT)

On the first day (day 0), larvae of the AB strain (purchased at the Center for Experimental Medicine in Lublin) the most commonly used strain in this type of research (30 dpf, 10 mm long; n = 40/group), were fed a high-fat diet in the form of 0.1% egg yolk (13, 14, 20–23). On the second day (day 1), larvae from the control group were starved, while group F1 was incubated with the foodstuff F1 – formulation 1 (250 μg/g body weight), group F2 with the foodstuff F2 – formulation 2 (250 μg/g body weight), and group F3 with the foodstuff F3 – formulation 3 (250 μg/g body weight). Visceral fat tissue was measured before and after fasting (control group) or exposure to the three formulations (F1, F2, F3) using Nile Red (NR) staining according to the method described by Zang et al. (day 3) (24). The NR signal was recorded using a fluorescence microscope.

Fluorescence images of zebrafish were acquired under identical imaging conditions (constant exposure time, gain, and illumination) and saved in TIFF format. Quantitative analysis was performed using an approach analogous to ImageJ. Prior to analysis, images were converted to 8-bit format and separated into RGB channels. Fluorescence intensity was quantified in the dominant emission channel (red). Background signal was excluded by restricting analysis to pixels corresponding to fish tissue using a binary body mask. Regions of interest (ROIs) were defined within a standardized ventral abdominal region using fixed anatomical landmarks, as indicated in reference images. The same ROI template (identical size, shape, and anatomical position) was applied consistently across all samples and experimental groups. ROI placement was therefore independent of fluorescence intensity and not adjusted based on signal distribution. Fluorescence measurements were performed as mean pixel intensity within each ROI after masking. To further ensure consistency, only the intersection of the ROI and the fish body mask was analyzed, eliminating any background contribution. All image processing and quantification were performed under blinded conditions with respect to experimental group allocation. For each experimental group, data are presented as mean ± standard deviation (SD), where SD reflects inter-individual variability (*n* = 4 per group). Statistical comparisons between groups were performed using an unpaired two-tailed Student’s t-test, with *p* < 0.05 considered statistically significant.

#### 2.2.5 Diet-induced obesity (DIO)

Standard fish food was enriched with 10% of the food in three different formulations F1, F2, F3. The tested formulations were ground to a granule diameter of 700 μm. Adult zebrafish (3 mpf – months post-fertilization, *n* = 40/group; purchased at the Center for Experimental Medicine in Lublin, Poland) were divided into five groups: control group was fed a standard diet for 3 weeks and from the second week, artemia at 5 mg/fish/day; group DIO (positive control) was fed a standard diet for 3 weeks and from the second week, artemia at 60 mg/fish/day to induced obesity. Groups DIO + F1 (F1 formulation), DIO + F2 (F2 formulation), and DIO + F3 (F3 formulation) were fed a diet containing the tested food for 3 weeks, and from the second week, additionally artemia at 60 mg/fish/day (23). Fish received approximately 6 mg/fish of experimental feed daily, administered once per day 30 min before artemia feeding to ensure consumption of the tested diet. Since the experimental feed contained 10% of the tested formulation, each fish received approximately 600 μg of formulation daily, corresponding to an estimated intake of approximately 250 μg of active compounds per fish per day. Before feeding and on the last day of the experiment, the body weight was measured. Glucose level, total cholesterol (LabAssay™ Cholesterol FUJIFILM Wako Pure Chemical Corporation, Japan) and triglycerides (LabAssay™ Triglyceride, FUJIFILM Wako Pure Chemical Corporation, Japan) were assessed using commercial kits. Before feeding and on the last day of the experiment, animals were anesthetized by placement in 40 mg/L ethyl 3-aminobenzoate methanesulfonate tricaine (until loss of equilibrium and absence of response to gentle tactile stimulation typically within 2–5 min) and then their body weight was measured (24). In the third week of the experiment, the animals were fasted for 24 h, and after blood sample collection (25) (glycemia and lipid profile assessment), they were euthanized by overdose of tricaine methanesulfonate (300 mg/L) according to Directive 2010/63/EU. Death was confirmed by cessation of opercular movements, absence of cardiac activity, and lack of reflex responses.

#### 2.2.6 Histology

After dissection, the animals were fixed in 4% paraformaldehyde, embedded in paraffin blocks, and sectioned using microtome. To assess fat distribution, sections of anatomically comparable areas of subcutaneous and visceral tissue were stained with hematoxylin–eosin and then assessed using a light microscope. The number of subcutaneous and visceral adipocytes per field of view and their area was manually counted for each individual (23).

#### 2.2.7 Statistical analysis

Shapiro–Wilk’s W test was used to assess data normality. Normally distributed data were analyzed using one-way analysis of variance (ANOVA) followed by Tukey’s post-hoc multiple comparisons test or by an unpaired Student’s *t*-test for comparisons between two groups. Data are presented as mean ± standard deviation (SD). Survival analysis was performed using the Kaplan–Meier method. The sample size was selected to ensure adequate statistical power. For the experimental design consisting of five groups with 40 fish per group (total *n* = 200), an *a priori* power analysis for one-way ANOVA at *α* = 0.05 indicated a statistical power of at least 0.80 to detect a medium effect size (Cohen’s *f* ≈ 0.25). Statistical analyses were conducted using GraphPad Prism software (version 10.5.0). Differences were considered statistically significant at *p* < 0.05.

## 3 Results

### 3.1 The effects of complex of active substances on zebrafish embryo/larvae development

#### 3.1.1 Embryos

**Figure 1:**
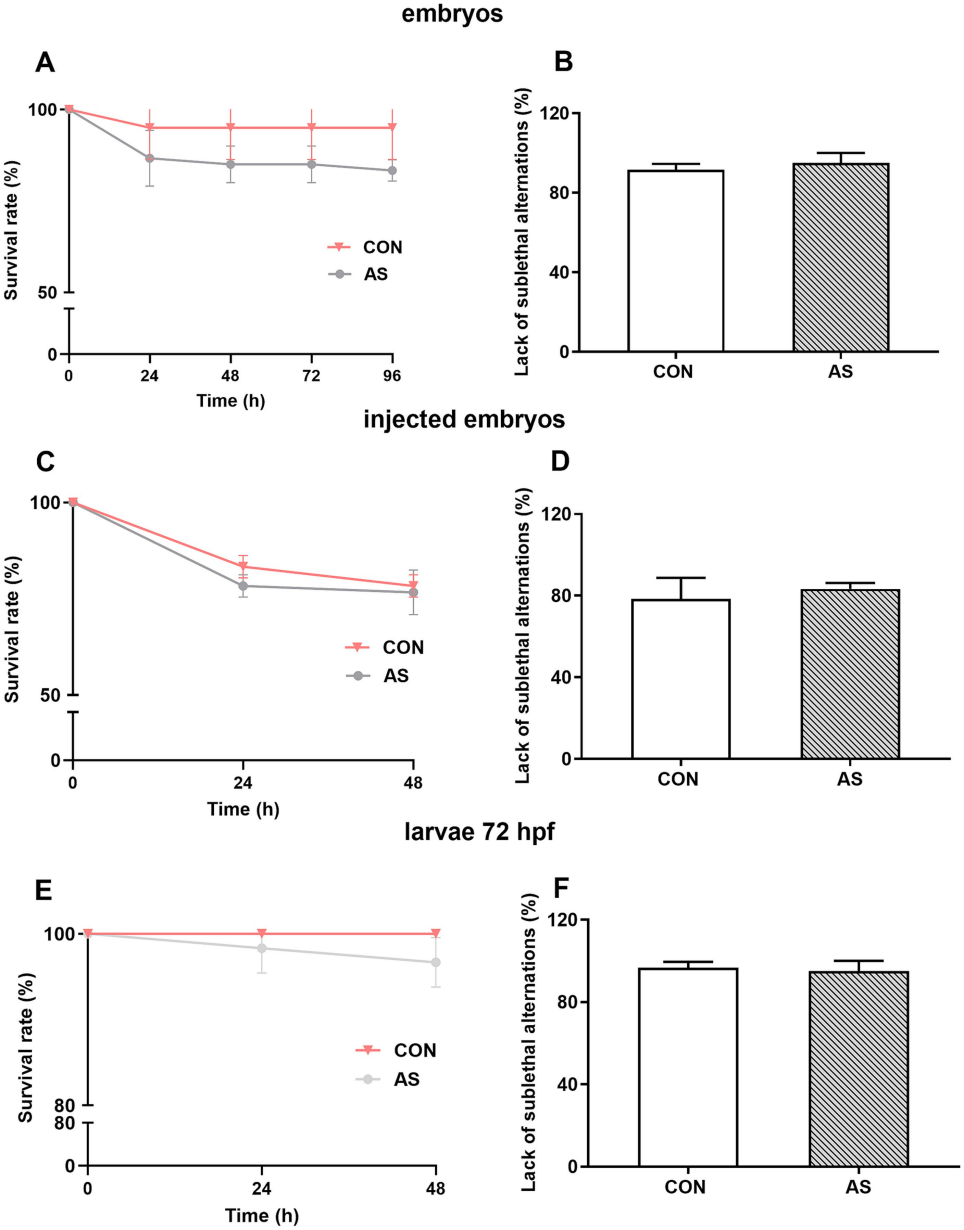
Survival rate **(A,E)** and incidence of sublethal alterations **(B,F)** in embryos (0–2 hpf) and larvae (72 hpf) incubated with 100 μg/mL complex of active substances (AS). Survival rate **(C)** and incidence of sublethal alterations **(D)** of AS injected embryos. Data are presented as mean ± SD.

The survival and early embryonic development were evaluated at 24, 48, 72, and 96 h after exposure to the 100 μg/mL complex of active substances (AS). No significant differences in embryo of 0–2 hpf survival were observed between the control group and embryos exposed to the AS (Figure 1). The occurrence of malformations, including pericardial and yolk sac oedema, tail curvature, and spinal scoliosis, was not significantly altered in any of the tested groups compared with the control (Figure 1). Survival and developmental parameters were also analyzed in the injected embryos at 24 and 48 h after exposure to the AS (8 nL). No significant differences in survival were detected between E3-treated controls and embryos exposed to the AS (Figure 1). The frequency of malformations did not differ significantly between treated groups and the control group (Figure 1).

**Figure 2:**
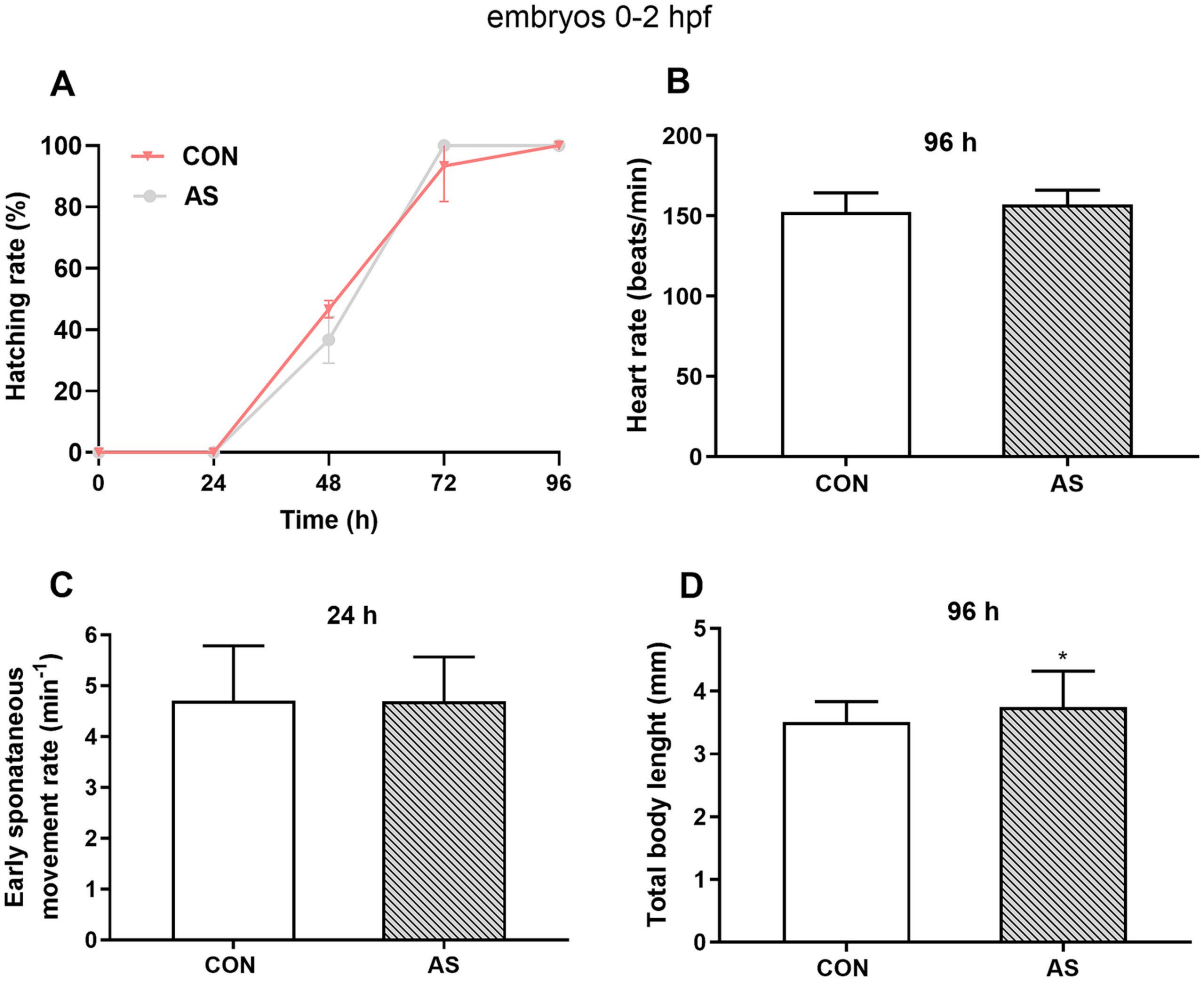
Effects of AS (complex of active substances) 100 μg/mL on zebrafish embryonic development and physiological parameters. Hatching rate of embryos exposed to control (CON) and AS over time (0–96 h post-fertilization, hpf) **(A)**. Heart rate measured at 96 hpf **(B)**, early spontaneous movement rate assessed at 24 hpf **(C)**, and total body length measured at 96 hpf **(D)**. Data are presented as mean ± SD. *p < 0.05 vs. CON.

The embryo hatching rate was assessed at 48 and 72 hpf by counting the number of larvae outside the eggshell, as hatching normally takes place within this time window. In the control group, approximately 46% of embryos hatched at 48 hpf and about 93% at 72 hpf. Exposure to the AS did not significantly affect the hatching rate compared to the control group (Figure 2). Early spontaneous movements were recorded after 24 h of incubation. In control embryos, side-to-side alternating contractions reached 4.7 ± 1.0 bends/min at 24 h. Similar bending frequency was observed in all treatment groups, indicating no effect of the AS on early spontaneous activity (Figure 2). Cardiac function, expressed as heart rate (HR), was not altered in embryos exposed to AS compared to the control at 96 h of incubation. Interestingly, embryos treated with the AS showed accelerated development, reflected by an increased total body length at 96 h of incubation (Figure 2).

#### 3.1.2 Larvae

The safety of the AS was further assessed in 72 hpf larvae, at the time selected based on a previous study (26). No mortality was observed in the untreated control group throughout the assay. Similarly, incubation with AS (100 μg/mL) did not result in significant larval mortality at any time point compared to the control group (Figure 1). During morphological evaluation, no significant malformations or developmental abnormalities were observed in the treated group compared to the control (Figure 1).

### 3.2 The influence of the tested formulations on the body weight, glucose level and lipid profile of fish with induced obesity

**Figure 3:**
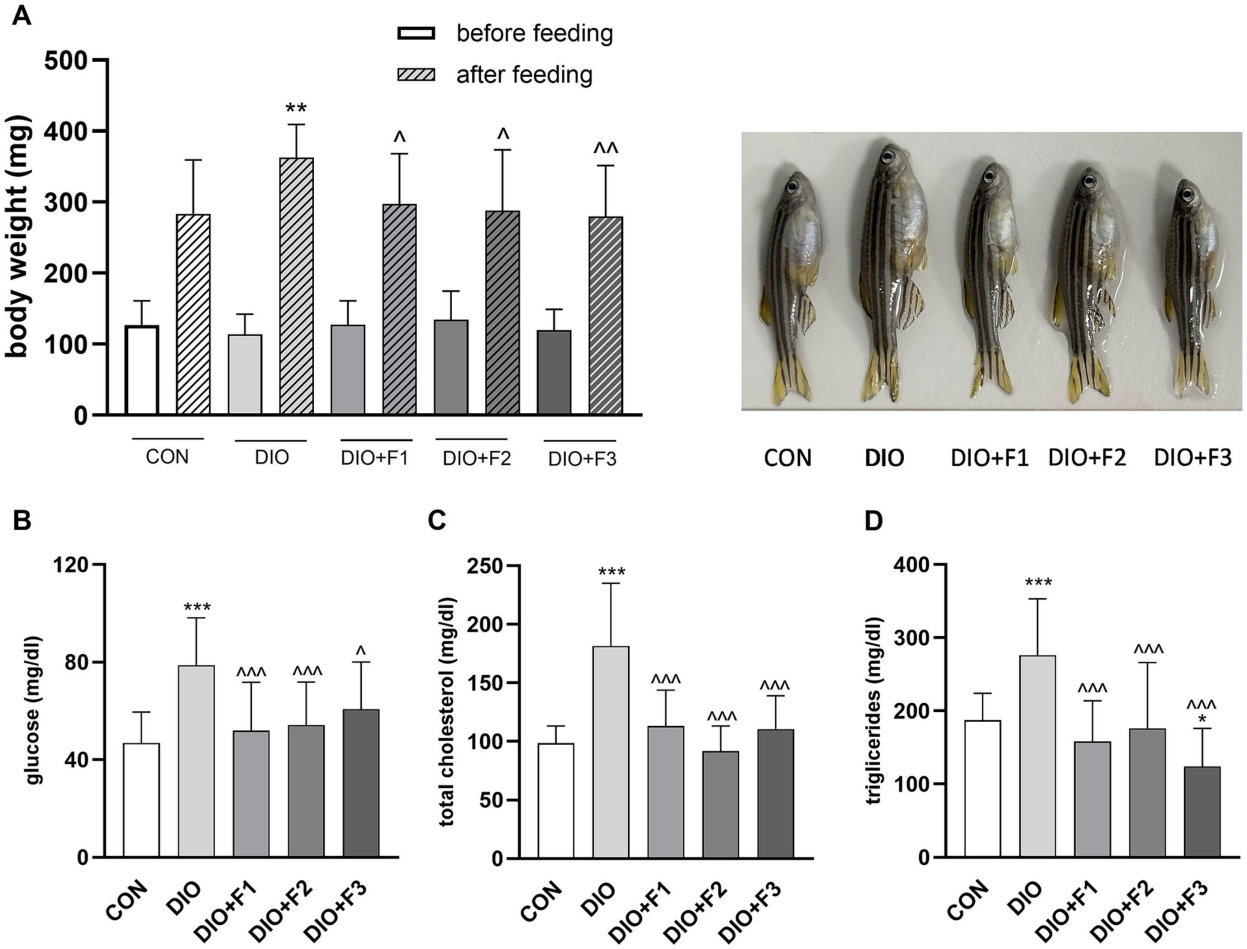
Effects of F1, F2, and F3 supplementation on body weight and metabolic parameters in zebrafish. Body weight measured before and after feeding in control (CON), diet-induced obesity (DIO), and DIO supplemented with F1, F2, or F3 **(A)**. Representative images of zebrafish from each group are shown on the right. Blood glucose levels **(B)**, total cholesterol levels **(C)**, and triglyceride levels **(D)**. Data are presented as mean ± SD *p < 0.05, **p < 0.01, ***p < 0.001 vs. CON; ^p < 0.05, ^^p < 0.01, ^^^p < 0.001 vs. DIO.

The foodstuffs in the three used formulations resulted in a significant reduction in body weight in animals from group DIO + F1 (297.4 ± 70; ^*p* < 0.05), DIO + F2 (288.1 ± 85; ^*p* < 0.05), and DIO + F3 (279.9 ± 71; ^^*p* < 0.01), compared to the DIO group (362.5 ± 46). They also significantly reduced fasting glucose levels (Figure 3A). The DIO group exhibited a marked increase in glucose concentration (78.8 ± 19 mg/dL; ***p < 0.001) compared to the control group (46.8 ± 12 mg/dL). Intervention with F1 (51.9 ± 19 mg/dL; ^^^*p* < 0.001 vs. DIO), F2 (54.3 ± 17 mg/d; ^^^*p* < 0.001 vs. DIO), and F3 (60.8 ± 19; ^p < 0.05 vs. DIO) formulations resulted in a reduction of glucose levels compared to the DIO group (78.8 ± 19 mg/dL). The DIO + F1 and DIO + F2 groups showed comparable decreases, with glucose values returning close to control levels (Figure 3B).

Total cholesterol level was markedly elevated in the DIO group (***p < 0.001) compared to the control group. Supplementation with F1, F2, and F3 led to a significant reduction in cholesterol concentrations (113.2 ± 30; 91.8 ± 30; 110 ± 28; ^^^*p* < 0.001, ^^^*p* < 0.001, ^^^*p* < 0.001 vs. DIO, respectively) (Figure 3C). Triglyceride level significantly increased in the DIO group (***p < 0.001) compared to the control. Supplementation with F1, F2, and F3 significantly reduced triglyceride concentrations (158.2 ± 55; 176.3 ± 89; 124.3 ± 51 ^^^*p* < 0.001, ^^^*p* < 0.001, ^^^*p* < 0.001 vs. DIO, respectively) (Figure 3D). The results indicate that the tested foodstuffs have a beneficial effect on glycemia, lipid profiles, and body weight.

### 3.3 The influence of the tested formulations on the fat tissue content in zebrafish

**Figure 4:**
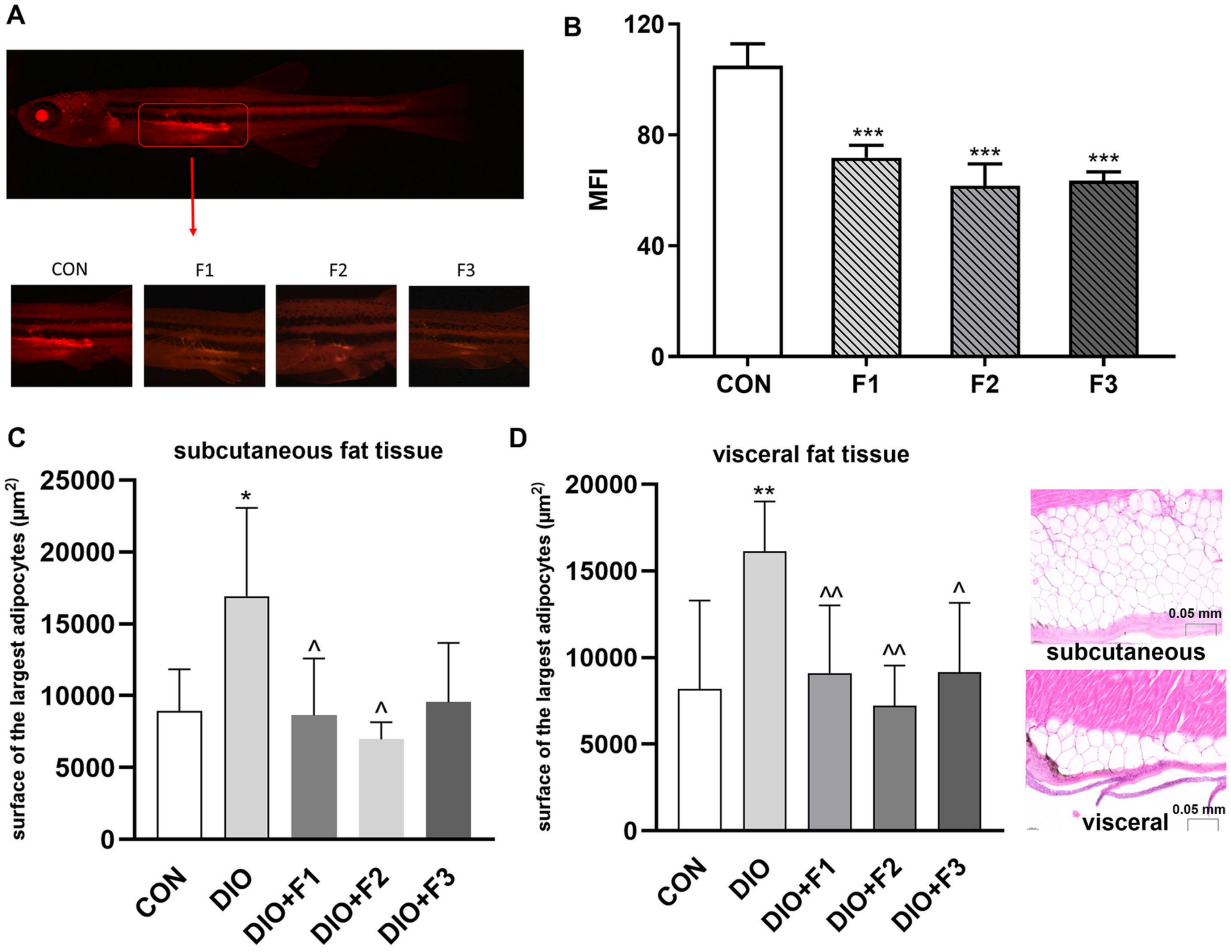
Effects of F1, F2, and F3 treatment on lipid accumulation and adipocyte morphology. Representative fluorescence image of a zebrafish larva showing lipid accumulation (red signal) **(A)**. Quantification of mean fluorescence intensity (MFI) indicating lipid levels in CON and F1, F2, and F3-treated group **(B)**. Surface area of the largest adipocytes in subcutaneous fat tissue **(C)** and visceral fat tissue **(D)** across experimental groups: control (CON), diet-induced obesity (DIO), and DIO supplemented with F1, F2, or F3. Representative histological images of subcutaneous and visceral adipose tissue are shown on the right. Data are presented as mean ± SD *p < 0.05, **p < 0.01, ***p < 0.001 vs. CON; ^*p* < 0.05, ^*^p* < 0.01 vs. DIO.

Quantitative analysis of fluorescence within the abdominal ROI revealed that the control group exhibited the highest signal intensity (104.9 ± 7.8). All experimental groups showed a reduction in fluorescence relative to control: F1 (71.7 ± 4.5), F2 (61.6 ± 7.9), and F3 (63.5 ± 3.2). This corresponds to a decrease of approximately 32% in F1, 41% in F2, and 40% in F3 relative to the control group. Statistical analysis confirmed that the reduction in fluorescence was significant for all experimental groups compared to control (F1, F2, and F3 ***p < 0.001; ***p < 0.001; ***p < 0.001 vs. CON). Overall, these results demonstrate a consistent and significant decrease in fluorescence intensity within the abdominal region across all treated groups, with the strongest effect observed in F2 and F3 (Figure 4A). Using the histopathology technique, the surface area of the largest adipocytes was assessed in both subcutaneous and visceral adipose tissue. In subcutaneous adipose tissue, the DIO group (*p < 0.05) exhibited a significant increase in adipocyte surface area compared with the control group. Supplementing the diet with F1 (^*p* < 0.05), F2 (^*p* < 0.05), and F3 attenuated this effect and showed reduced adipocyte size relative to DIO. A similar trend was observed in visceral adipose tissue. The DIO group (**p < 0.01) showed a significant elevation in adipocyte surface area compared to the control. Enrichment of the diet with F1 (^^*p* < 0.01), F2 (^^*p* < 0.01), and F3 (^*p* < 0.05) significantly decreased adipocyte size relative to DIO. These findings indicate that diet-induced obesity promotes adipocyte hypertrophy in both fat depots, while the tested interventions mitigate this effect. (Figures 4B,C).

## 4 Discussion

Obesity is characterised by enlarged visceral fat and alterations in glucose and lipid metabolism (1). New treatment approaches should cover all these challenges to provide the most prominent results. The present study evaluated the effects of new food formulations (F1, F2, and F3) on the development, metabolic parameters, and fat tissue content in a zebrafish model. The findings demonstrate that the tested foodstuffs are safe at early developmental stages and exert beneficial metabolic effects in an obesity model, suggesting their potential as functional dietary interventions.

A complex composed of multiple active ingredients (AS) was created and incorporated into three nutritional matrices (formulation F1, F2, F3). The complex contains quercetin, which exhibits well-documented antihypertensive effects, supports the optimization of lipid metabolism, improves endothelial function, and provides strong antioxidant protection (27). The formulation also includes flavonol - rutin, which acts synergistically with quercetin. It contributes to prolonged vascular protection, supports glycemic control by reducing fasting glucose levels, and further strengthens blood vessels, which is essential for the prevention of microcirculatory disorders (28). The complex was further enriched with betaine (trimethylglycine), which reduces homocysteine levels—an amino acid associated with metabolic disturbances. Moreover, betaine facilitates the transport of fats from the liver, helping to prevent hepatic steatosis, and supports improvements in the muscle-to-fat ratio, thereby enhancing insulin sensitivity (29). Additionally, white mulberry extract standardized to 1-deoxynojirimycin (1-DNJ), as a natural *α*-glucosidase inhibitor, was added. 1-DNJ delays carbohydrate digestion in the intestine and attenuates the postprandial glycemic response. In addition to its effects on carbohydrate metabolism, mulberry-derived iminosugars influence lipid metabolism and inflammatory pathways, suggesting their potential supportive role in regulating metabolic disorders associated with obesity and impaired glucose homeostasis (30). The final component incorporated into AS is zinc citrate. Zinc is involved in numerous enzymatic processes related to glucose and lipid metabolism, as well as insulin signaling. Experimental and clinical studies have shown that adequate zinc intake is associated with improved metabolic control and modulation of lipid parameters, indicating its possible contribution to maintaining metabolic homeostasis (31).

First, the safety profile of the complex of active substances (AS) was confirmed in both embryos and larvae. Exposure to AS did not affect survival, hatching rate, spontaneous movement, or cardiac function, and no increase in developmental malformations was observed. These findings indicate that the AS is not toxic during early developmental stages. Interestingly, the AS was associated with increased body length at 96 hpf, which may suggest a stimulatory effect on growth processes. This observation requires further investigation to determine its biological relevance and underlying mechanisms. These findings are consistent with previous reports demonstrating a low toxicity profile of flavonoids in the *Danio rerio* model during early developmental stages. Both quercetin and rutin have been widely described as biologically active compounds with minimal embryotoxic effects at low to moderate concentrations, primarily exerting antioxidant and anti-inflammatory activities (32, 33). Similarly, betaine is generally recognized as a safe compound with important physiological functions, including its role as a methyl donor and osmoprotectant (34). Previous studies have shown that betaine supports cellular homeostasis, protects against environmental stress, and may positively influence growth and metabolic processes (35, 36). Interestingly, the increased body length at 96 hpf may suggest a potential stimulatory effect on growth-related processes. This observation could be linked to the known biological activities of flavonoids and methyl donors. In parallel, betaine, through its involvement in one-carbon metabolism, may influence epigenetic regulation and gene expression associated with growth pathways, including the insulin-like growth factor (IGF) axis (37). However, it should be emphasized that the observed increase in body length requires cautious interpretation. The literature reports both growth-promoting and neutral effects of these compounds, depending on concentration, exposure duration, and experimental conditions.

The adult zebrafish DIO model revealed significant metabolic improvements following administration of the tested foodstuffs. Overfeeding with artemia successfully induced an obesity-like phenotype, as evidenced by increased body weight and altered metabolic parameters. Supplementation with F1, F2, or F3 resulted in significant reductions in body weight, glucose, triglycerides, and total cholesterol levels compared to the obese group. These results suggest that the tested formulations may modulate key pathways involved in energy metabolism, glucose homeostasis, and lipid regulation. The results of our study are consistent with previous reports on dietary interventions in obese zebrafish models, where treatments such as natural phytoconstituents olivetol, orange juice, cinnamon, green tea extract, and Campari tomato have been shown to prevent body weight gain (20, 38–41). In the model of obese zebrafish, natural approaches to obesity prevention resulted in reduced TG (17, 20, 24, 40, 42) and cholesterol levels (24, 43). However, a decrease in TG level was not always accompanied by a reduction in cholesterol level (34) or there was only a significant change in cholesterol without affecting TG level (43). The data regarding glucose levels in the treatment of the obese zebrafish model is more limited. In one study, cinnamon supplementation significantly decreased glucose level to the values comparable to the control group (40), while green tea extract did not affect the fasting blood glucose levels (24), as well as resveratrol treatment (43).

All three tested formulations contained standardized bioactive compounds, including quercetin, rutin, and betaine, which are known to modulate lipid metabolism, improve insulin sensitivity, and reduce oxidative stress (36, 44, 45). Quercetin may act through pathways such as AMP-activated protein kinase (AMPK) activation and regulation of glucose uptake (44). In addition, the presence of 1-deoxynojirimycin (1-DNJ) derived from white mulberry extract is particularly relevant for glucose homeostasis. 1-DNJ is a well-characterized *α*-glucosidase inhibitor that delays carbohydrate digestion and reduces postprandial glucose levels, thereby contributing to improved glycemic control (46, 47). This mechanism may partly explain the significant reduction in glucose levels observed across all treatment groups. The formulations also contained substantial amounts of dietary fiber, including psyllium, inulin, and glucomannan (F1), as well as fiber-rich plant ingredients such as flaxseed, oat flakes, and legumes (F2 and F3). Dietary fibers are known to modulate gut microbiota composition, slow nutrient absorption, and improve lipid and glucose metabolism (48). Furthermore, the inclusion of polyphenol-rich fruits (e.g., bilberry, elderberry, rosehip, acerola) provides additional antioxidant and anti-inflammatory compounds, particularly anthocyanins and vitamin C, which have been associated with reduced adiposity and improved metabolic profiles (49). Other bioactive plant extracts, such as *Galega officinalis* L. (present in F2), have been traditionally linked to hypoglycaemic effects and are considered precursors in the development of antidiabetic therapies due to their influence on glucose metabolism (50). Similarly, spices and plant-derived compounds (e.g., turmeric, garlic) may contribute to the observed effects through anti-inflammatory and metabolic regulatory properties. The observed improvements in metabolic parameters likely result from synergistic interactions between multiple bioactive components, including flavonoids, methyl donors, *α*-glucosidase inhibitors, dietary fibers, and antioxidant-rich plant ingredients. Previous studies suggest that these bioactive compounds may influence pathways involved in energy balance, glucose homeostasis, lipid metabolism, and inflammation.

The observed improvement in metabolic outcomes are consistent with previous studies demonstrating that dietary components and bioactive compounds can influence metabolic processes in *Danio rerio* (12, 17, 51, 52). In particular, flavonoid-rich extracts, dietary fibers, and polyphenol-containing plant materials have been shown to modulate glucose and lipid metabolism in zebrafish and other vertebrate models, supporting their potential role in metabolic regulation (12, 14). These effects are often attributed to the combined action of antioxidant activity, modulation of energy balance pathways, and regulation of nutrient absorption. The reduction in glycemia and lipid levels observed in the present study may reflect metabolic effects previously associated with improved insulin sensitivity and reduced lipogenesis in other experimental models (53, 54). This interpretation is supported by previous studies reporting that quercetin may activate AMPK, suppress lipogenic gene expression, and enhance glucose uptake in peripheral tissues (44, 55). Similarly, betaine has been associated with improved insulin signalling and lipid homeostasis through its role in one-carbon metabolism and methylation-dependent gene expression regulation (34, 36). Future investigations should focus on elucidating the molecular pathways involved, including potential effects on insulin signalling cascades (e.g., PI3K/Akt pathway), lipid metabolism genes (such as SREBP-1c, PPARα, and PPARγ), and inflammatory processes, which are known to play a central role in metabolic regulation in zebrafish models. However, the present study did not include direct molecular analyses; therefore, the proposed mechanisms remain speculative and require confirmation by gene and protein expression studies.

In addition to systemic metabolic effects, the tested formulations significantly reduced visceral fat accumulation in *Danio rerio* larvae, as demonstrated by Nile Red staining. The decrease in lipid accumulation may reflect enhanced lipid mobilization, reduced lipid uptake, or alterations in adipocyte differentiation, processes that are known to be regulated by bioactive dietary components, including flavonoids and fibers. Given the relevance of visceral adiposity in the development of metabolic disorders, including insulin resistance and dyslipidemia, this effect is particularly important (56–58). Similar reductions in lipid deposition have been reported following exposure to dietary polyphenols and fiber-rich interventions in zebrafish, suggesting modulation of adipogenesis and lipid storage pathways (20, 41). *Palmaria mollis*, a popular red seaweed, was found to decrease the volume of visceral adipose tissues in DIO zebrafish, which was also confirmed in the mouse model (17). A similar outcome was observed after the green tea extract treatment (24, 42, 59), however, it did not affect the subcutaneous fat volume (59). White grape juice extract reduces fat accumulation in overfed zebrafish by modulating ghrelin and leptin expression (60).

## 5 Conclusion

Overall, the present study demonstrates that the tested formulations (F1–F3) exert a pronounced and multi-level beneficial effect on metabolic parameters in the adult *Danio rerio* diet-induced obesity model. Across all experimental groups, supplementation effectively counteracted the obesogenic effects of *Artemia* overfeeding, leading to significant reductions in body weight and improvements in glycemic and lipid profiles, including decreased glucose, triglyceride, and total cholesterol levels. Importantly, these metabolic benefits were accompanied by a marked reduction in visceral lipid accumulation in larvae, indicating that the formulations improved systemic biochemical markers and directly influenced lipid storage at the tissue level. This was further supported by Nile Red staining, which confirmed reduced fat deposition and suggested a functional impact on adipogenesis and lipid mobilization. The observed effects are likely attributable to the synergistic action of multiple bioactive components present in the formulations, including flavonoids (quercetin and rutin), methyl donors such as betaine, dietary fibers, and plant-derived antioxidants. These compounds are known to modulate key metabolic pathways involved in glucose homeostasis, lipid metabolism, oxidative stress, and inflammation, suggesting a multi-target mechanism of action. Although zebrafish (*Danio rerio*) represent a well-established vertebrate model with considerable translational relevance to human metabolic research, caution should be exercised when extrapolating these findings to humans due to inherent interspecies physiological differences. In conclusion, the results indicate that the tested food formulations may represent promising functional dietary interventions capable of improving metabolic health and reducing obesity-related phenotypes *in vivo*.

## Data Availability

The raw data supporting the conclusions of this article will be made available by the authors, without undue reservation.
